# Quantification of the Relative Importance of CTL, B Cell, NK Cell, and Target Cell Limitation in the Control of Primary SIV-Infection

**DOI:** 10.1371/journal.pcbi.1001103

**Published:** 2011-03-03

**Authors:** Marjet Elemans, Rodolphe Thiébaut, Amitinder Kaur, Becca Asquith

**Affiliations:** 1 Department of Immunology, Imperial College London, London, United Kingdom; 2 ISPED, Bordeaux Segalen University, Bordeaux, France; 3 INSERM U897 Epidemiology and Biostatistics, Bordeaux, France; 4 Division of Immunology, New England Primate Research Center, Harvard Medical School, Southborough, Massachusetts, United States of America; Utrecht University, Netherlands

## Abstract

CD8^+^ cytotoxic T lymphocytes (CTLs), natural killer (NK) cells, B cells and target cell limitation have all been suggested to play a role in the control of SIV and HIV-1 infection. However, previous research typically studied each population in isolation leaving the magnitude, relative importance and *in vivo* relevance of each effect unclear. Here we quantify the relative importance of CTLs, NK cells, B cells and target cell limitation in controlling acute SIV infection in rhesus macaques. Using three different methods, we find that the availability of target cells and CD8^+^ T cells are important predictors of viral load dynamics. If CTL are assumed to mediate this anti-viral effect via a lytic mechanism then we estimate that CTL killing is responsible for approximately 40% of productively infected cell death, the remaining cell death being attributable to intrinsic, immune (CD8^+^ T cell, NK cell, B cell) -independent mechanisms. Furthermore, we find that NK cells have little impact on the death rate of infected CD4^+^ cells and that their net impact is to increase viral load. We hypothesize that NK cells play a detrimental role in SIV infection, possibly by increasing T cell activation.

## Author Summary

The role of the immune response in controlling HIV infection, or its simian counterpart SIV, has not been fully elucidated. Different studies have provided evidence of a role for different components of the immune system. Unfortunately, the different components are usually studied in isolation, making it hard to determine their relative importance. Here we study three main immune cell populations, T, B and NK cells, in parallel and found that cytotoxic T cells play the largest role in the control of SIV infection, but were not responsible for the majority of infected cell death. Interestingly, we found indications that, although NK cells contribute to the killing of infected cells, the net impact of the NK response is to increase viral load.

## Introduction

During the early stage of infection with human or simian immunodeficiency virus type 1 (HIV-1, SIV-1) the number of circulating virus particles increases rapidly, typically doubling every 6–10 hours [1]–[4]. This is accompanied by the fast destruction of CD4^+^ T cells in the mucosa and to a lesser extent in the periphery [5]–[7]. Subsequently, in virtually all infected individuals, viral load starts to decline and falls to a relatively stable level or set point that is typically several orders of magnitude lower than the peak viral load [8]–[10]. What causes this reproducible and robust, yet ultimately incomplete, control of viral replication is still unclear. Understanding the factors that are naturally effective in controlling virus infection may be key to engineering practical and widely applicable treatment for control of HIV-1 infection. Some of the main factors that have been postulated to play a role are CD8^+^ cytotoxic T lymphocytes (CTLs), natural killer (NK) cells, B cells and target cell limitation.

SIV/HIV-1-specific CD8^+^ cytotoxic T cells are widely considered to help control SIV and HIV-1. Observations supporting a role for CD8^+^ T cells in the containment of immunodeficiency virus are i) the temporal association between the appearance of HIV- or SIV-specific CD8^+^ T cell responses and the post-peak decline in viral load [11]–[13], ii) the significant association of particular MHC-class I alleles with protection from HIV-1 disease progression [14], iii) the dramatic increase in SIV viral load after in vivo depletion of CD8^+^ T cells [15]–[17], and iv) the existence of multiple viral mechanisms to evade the CTL response, including down-regulation of HLA class I A and B molecules from the surface of infected cells [18] and evolution of mutated forms of viral epitopes that escape CD8^+^ T cell surveillance [19]. However, numerous studies have reported that CD8^+^ T cells are poorly functional in HIV-1 infection [20], possibly due to exhaustion [21], [22], CD4^+^ depletion and/or an immature phenotype [23], [24]. For instance, the fraction of PD-1^+^ CD8^+^ T cells is more than two-fold higher in HIV-specific compared to the total population [21] and the proportion of CD27^+^ cells is above 50% in HIV-specific T cells compared to well below 50% in CMV-specific T cells [23]. Furthermore, quantification of the selection pressure exerted by CTL responses in HIV-1-infected individuals in vivo suggested that only a minority (20–40%) of productively-infected CD4^+^ cell death in chronic/late primary infection is attributable to CTL killing [25].

To control their cytotoxic activity, NK cells possess two types of surface receptors: activating receptors, whose ligands include certain stress molecules, and inhibitory receptors, which bind MHC class I molecules. Integration of signals from these receptors determines NK lytic function [26]. Evidence for a role of NK cells in the control of HIV-1 infection is comparable to the evidence for CD8^+^ T cell control. Several studies [27], [28] report an expansion of the cytolytic CD56-dim NK cell subset in acute HIV-infection. Depletion of cytolytic CD16^+^ NK cells, although short-lived and incomplete, showed a trend towards higher levels of SIV replication in NK cell-depleted monkeys compared to control monkeys [29]. Furthermore, HIV escapes NK cell recognition by preventing the down-regulation of HLA-C/E molecules [18] and restricts the up-regulation of ligands for activating NK cells receptors like MICA, ULBP1 and 2 [30] and NKp44L [31]. Lastly, an activating NK cell receptor (KIR3DS1), in combination with its putative ligand (HLA-B Bw40-80I) has been shown to be associated with slower HIV-1 disease progression [32] while in HIV-exposed uninfected individuals a higher prevalence of KIR3DS1 homozygosity was found [33]. This may be caused by preferential activation and expansion of KIR3DS1- or KIR3DL1-expressing NK cells and subsequent inhibition of HIV-1 replication [34]–[37]. However, O'Connell *et al* [38] did not find that KIR3DS1 was overrepresented in a cohort of Elite Suppressors (ES) and concluded that strong NK cell-mediated inhibition of viral replication is not necessary for the control of HIV-1 in all ES. Moreover, various studies found a decline in number and lytic function of NK cells from early in infection [39]–[41].

Evidence for the role of B cells includes the rapid and continuous *in vivo* evolution of Env, the primary target for neutralizing antibodies [42], [43] and a negative correlation between the level of neutralizing Abs and viral load during chronic [44], but not acute infection [45]. Additionally, depletion of B cells in rhesus macaques has demonstrated that humoral immune responses may help to control viraemia during the immediate post-acute phase of infection [46], [47]. However, Gaufin *et al* [48] found no significant effect of B cell depletion on viral load in SIV infection in African Green Monkeys, concluding that humoral immune responses play only a minor role in the control of viral replication in the natural host. Furthermore, HIV-associated premature exhaustion of B cells has been described and may contribute to poor antibody responses against HIV [49].

An alternative to immune control is target cell limitation [50]. Using mathematical modelling, Phillips [50] demonstrated that the post-peak decline of virus load could be due to the loss of CD4^+^ T cells as targets for viral replication. Experimental work supporting a role for target cell limitation includes studies describing post-peak viral decline in the absence of a specific immune response [51]–[53]. Further support is provided by studies that found a reduction in set point viral load when the immune system was artificially suppressed [54]–[57] or an increase in set point viral load when target cell levels were increased, for instance by IL-2 [58], [59] or vaccination [60]–[65]. However, more recent theoretical work has questioned the importance of target cell limitation [66], [67] and Zhang *et al* [68] found an increase in activated CD4^+^ T cells in the lymphatic tissues of acutely SIV-infected rhesus macaques, even as levels of virus in plasma fell.

In short, studies of each of these factors, namely CD8^+^ T cells, NK cells, B cells and target cell limitation, have found evidence for their anti-viral activity. But the magnitude, relative importance and in vivo relevance of these effects is unclear. At least part of the problem is that studies have tended to focus on each factor in isolation. The aim of this work was to quantify the relative importance of CD8^+^ T cells, NK cells, B cells cytolytic activity and target cell limitation in controlling acute SIV viraemia.

## Results

The factors determining early SIV viral load in 17 experimentally infected rhesus macaques were investigated using three different approaches. First, simple empirical models were used to assess the temporal (“Granger–causal”) relationship between viral load, target cells and immune populations within each animal. Secondly, different mechanisms of viral control were studied using mechanistic dynamical models. Finally, we assessed the ability of each cell population to predict viral load variation across the cohort. The first and the third approach make no assumption about the mechanism of target cell and immune cell action. In the second, mechanistic, approach we investigated different, plausible modes of action.

### Granger causality

#### Combination of CD4^+^ and CD8^+^ T cells gives the best prediction of viral load

Granger causality is a weak definition of causality originally developed in economics [70] and now used in all branches of science. If an explanatory variable is able to help predict the *future* values of a dependent variable then the explanatory variable is said to “Granger-cause” the dependent variable.

**Figure 1:**
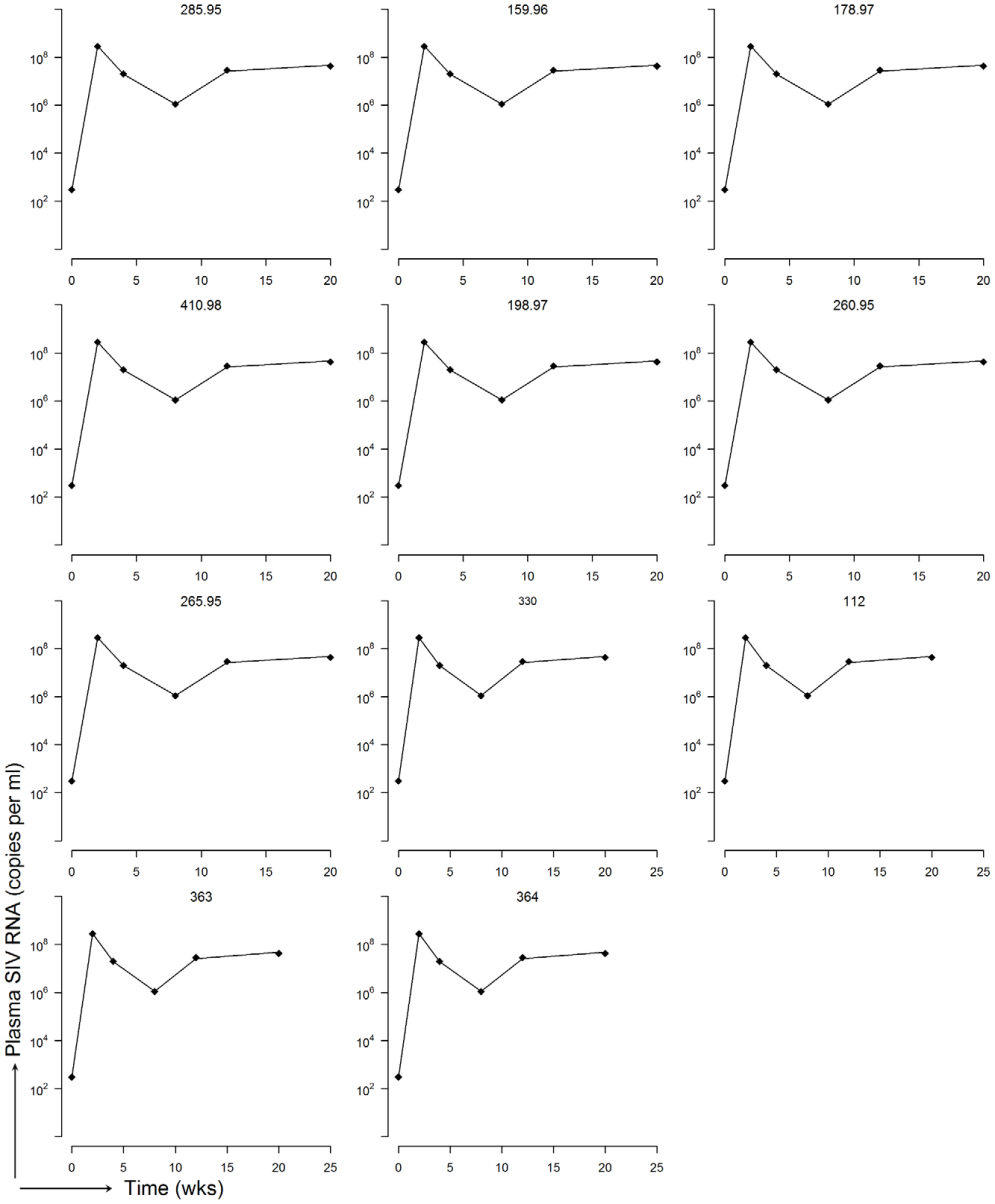
Experimental data and fits of empirical model (Granger-causality). Experimental viral load and viral load predicted by a regression of past VL, past CD4^+^ T cells and past CD8^+^ T cells for each of the 11 datasets.

We investigated the Granger-causal determinants of viral load. For each of the immune cell populations of interest (CD8^+^ T cells, NK cells, B cells) we asked whether that population, alone or in combination with CD4^+^ target cells, or target cells alone, was a significantly better predictor of future viral load than past viral load alone. Importantly, Granger-causality makes no assumption about the mechanism of action i.e. it is essentially a hypothesis-free method. We used a population approach through a Bayesian framework which provides increased power to distinguish between different models. Results are shown in Table 1. We found that the best fit, indeed the only model to show a substantial improvement over viral load alone, was a model including CD8^+^ T cells and CD4^+^ T cells (Figure 1). Weak support was found for a model with CD4^+^ cells alone. There was no support for models containing NK cells or B cells either alone, or in combination with CD4^+^ cells.

**Table 1:** Granger causality model inference.

	Dependent	Predictors	ΔDIC
A	V_t_	V_t-τ,_ T_t-τ_	−2.6
	V_t_	V_t-τ,_ CD8_t-τ_	−20.5
	V_t_	V_t-τ,_ NK_t-τ_	−18.5
	V_t_	V_t-τ,_ B_t-τ_	−5.4
	**V_t_**	**V_t-τ,_ T_t-τ,_ CD8_t-τ_**	**5.8**
	V_t_	V_t-τ,_ T_t-τ,_ NK_t-τ_	−13.9
	V_t_	V_t-τ,_ T_t-τ,_ B_t-τ_	−22.0
B	CD8_t_	CD8_t-τ,_ V_t-τ_	1.6
	NK_t_	NK_t-τ,_ V_t-τ_	−0.3
	B_t_	B_t-τ,_ V_t-τ_	3.4
	T_t_	T_t-τ,_ V_t-τ_	3.6

Comparison of A) Do any of the lymphocyte populations Granger-cause viral load? The Deviance Information Criterion (DIC) of the simplest model (past viral load predicts future viral load) was compared with the DIC of alternative models in which past viral load and a range of different lymphocyte populations (alone or in combination as specified in the table) were predictors of future viral load. B) Does viral load Granger-cause the lymphocyte populations? The analysis was reversed and the DIC of the listed models is compared with the DIC of models including only one of the lymphocyte populations as predictor (i.e. without viral load). ΔDIC is defined as DIC of the simplest model minus the DIC of the more complex model. As a rule of thumb we consider strong support for the simplest model when ΔDIC is <2, more support for the more complex model when ΔDIC<7 and strong support for the complex model when ΔDIC>10 [90]. V_t_: viral load at time t. T_t_: CD4^+^ T cells at time t. CD8_t_: CD8^+^ T cells at time t. NK_t_: NK cells at time t. B_t_: B cells at time t. The only model to show a large improvement over the simplest model with past viral load only was a model with past CD8^+^ T cells and target cells (in bold above).

Reversing the process to test whether viral load causes CD8^+^ T cell dynamics we found that although there was a trend, viral load was not a significant predictor of future numbers of CD8^+^ T cells. There was some support for a role of viral load in the prediction of CD4^+^ T cells (Table 1B).

We conclude that CD8^+^ T cells and CD4^+^ target cells are Granger-causal determinants of SIV viral load. Importantly, this result did not follow trivially from viral load driving CD8^+^ T cell expansion as viral load was not a strong predictor of CD8^+^ T cells. B cells and NK cells are not causal predictors of viral load, even with this weak definition of causality.

### Mechanistic models

#### CD8^+^ immune control explains viral dynamics best

Mechanistic models are widely used in biology to investigate population dynamics. We used the approach of Regoes *et al* ([66], Methods) to test whether immune control by CD8^+^ T cells, NK cells, B cells or target cell limitation accounts best for the viral dynamics in acute SIV infection. Again, we implemented the fitting in a Bayesian framework in which models were fitted to all animals simultaneously. Although, this naturally results in less good fits to data from each individual animal it gives increased power to distinguish between models and should be better to predict the dynamics in new animals.

**Figure 2:**
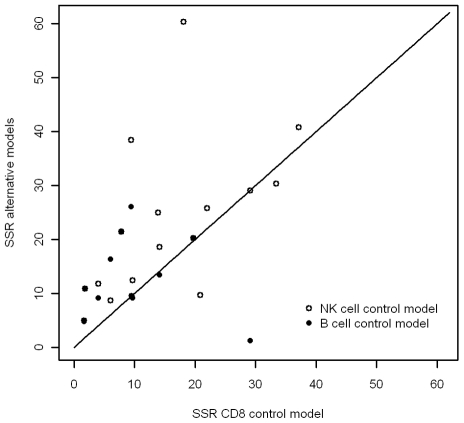
Difference between model predictions and observed data (sum of squared residuals). The x axis is the deviation of the predictions of a CD8^+^ T cell model from the data; the y axis is the deviation for the two alternative models, NK cell control model, and B cell control model. All models have an equal number of parameters The line is the line of equal SSR. It can be seen that the match between the prediction and the observed data is best (lowest SSR) for the CD8^+^ T cell model (p = 0.17 when compared to B cells, p = 0.02 when compared to NK cells).

**Figure 3:**
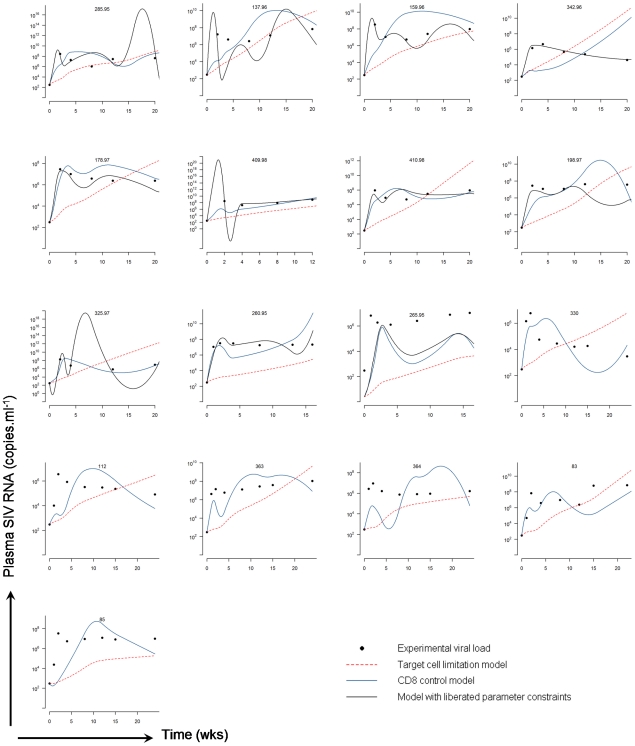
Experimental data and mechanistic model fits. Experimental viral load and viral load predicted by the target cell limitation model and CD8 control model. B cell data was missing for 6 of the datasets and so the model with liberated parameter constraints could not be fitted for these animals. The oscillations in the predicted viral load are due to variation in the experimental data underlying the empirical functions describing the dynamics of target and immune cell populations. Smoothing of the input data reduces the fluctuations. Note: all datasets were fitted simultaneously, not individually.

We found that the CD8^+^ T cell control model best describes the experimental viral load data. Figure 2 shows that the fit of the CD8 control model, represented by the sum of squared residuals, is consistently better than the fit of any of the other three models. The target cell limitation model yields poor fits (Figure 3) and, compared to the CD8 control model, gains no support (Table 2A). The NK cell control model also performs poorly. The fit of the B cell control model, although better than the target cell and NK models, does not compete with the CD8^+^ T cell control model. In the CD8^+^ T cell control model described above, CD8^+^ T cells are assumed to mediate control via a lytic mechanism. If CD8^+^ T cells are assumed to operate via a non-lytic mechanism then this provides a further improvement in the fits (Table S1), and confirms the importance of CD8^+^ T cells in predicting HIV dynamics. The oscillations in the predicted viral load are due to the large variation in the experimental data underlying the empirical functions describing the dynamics of target and immune cell populations. Smoothing of the input data reduces the fluctuations.

**Table 2:** Inference mechanistic models.

A.	ΔDIC
**CD8** ^+^ **T cell immune control**	**42.5**
NK cell immune control	0.7
B cell immune control	23.3

B	
Ki67^+^ target cell limitation	−3.2
Ki67^+^ CD8^+^ T cell control	−27.6
Env- and Gag- specific T cell control	−11.3
Ki67^+^ NK cell control	−7.9
Ki67^+^ B cell control	−14.3

C	
CD8 and NK cell immune control	−9.3
CD8 and B cell immune control	2.5
B and NK cell immune control	−21.4
CD8, NK and B cell immune control	−16.8

ΔDIC calculated as A) DIC target cell limitation model minus DIC immune control model, B) DIC immune control model minus DIC Ki67^+^ immune control model or DIC Env and Gag-specific immune control model and C) DIC CD8 control model minus DIC immune control models combining two or three immune populations. As a rule of thumb we say we cannot distinguish between two models when we find ΔDIC <2, if ΔDIC is between 3 and 7 there is some support for the 1^st^ model but the 2^nd^ model is clearly better, if ΔDIC is greater than 10 essentially no support is found for the 1^st^ model [90]. The model that predicts the experimental data best is the CD8^+^ T cell immune control model (in bold above).

No improvements in fit were found when we repeated the simulations using the Ki67^+^ fraction of each cell population (Table 2B). Similarly, repeating the simulations with Env- and Gag-specific CD8^+^ T cells also did not yield an improvement in the fits.

Combination of two or three immune cell populations does not substantially improve the prediction of viral dynamics for most combinations (Table 2C). Only the combination of CD8^+^ T and CD20^+^ B cells in one model gets some support, but it is only marginally better than the simpler model with CD8 cell control only. When applying the least squares regression approach to models with two or more effector populations, searching for point estimates instead of posterior distributions, we found that in most cases all killing rates except for CD8 cells were estimated to be zero (data not shown).

#### Quantification of the impact of different immune effector populations

Next, in a model in which each of the immune effector cells was allowed to exert an antiviral effect via infected cell lysis, we quantified the contribution of the different immune cell populations to productively infected cell death (Methods, Table 3). We found that, in this model, the main causes of infected CD4^+^ T cell death are the intrinsic (CD8, NK and B-independent) death rate, accounting for almost half of infected cell death (47%), and CD8^+^ T cell killing (42%). B cells and NK cells play only a minor role in the death of productively infected cells (both approximately 6%).

**Table 3:** Contribution to total cell death of intrinsic death rate and the different immune populations.

	Average (SD)
Intrinsic death rate	46.8% (16.9)
CD8^+^ T cell killing	41.8% (12.7)
B cell killing	5.7% (3.5)
NK cell killing	5.7% (4.2)

Contribution averaged over different time points and all animals is given, standard deviation in brackets.

These results support the findings of Grangers causality, identifying CD4^+^ and CD8^+^ T cells as the most important factors in the containment of viral replication, giving no substantial support to NK and B cell-mediated control of SIV-infected cells.

#### Possible alternative role for NK cells in SIV-infection

Next we asked, in a simple correlation analysis, how much of the variation in viral load across the cohort at time t could be explained by variation in the various factors at the previous time point (see Methods, partitioning of R2, and Table 4). Surprisingly, given the minor role which we had found for them in killing infected cells, NK cells predicted a high proportion of variation in future viral load (15.3%).

**Table 4:** Contribution to R^2^.

	Partitioned R^2^
VL	55.3
CD4^+^ T cells	17.6
CD8^+^ T cells	7.5
B cells	4.4
NK cells	15.3

The partitioned R^2^ indicates how much of the total variation in future viral load is attributable to the various factors.

Based on this large contribution of NK cells to explaining experimental variance in viral load but not killing, in combination with a consistent positive effect of NK cells on viral load found in the Granger regression when including all three immune populations (data not shown), we decided to test how releasing the parameter constraints on the killing rates of CD8^+^ T cells, NK cells and B cells would affect the prediction of viral load in the mechanistic model. Because no quantitative information is available regarding a possible positive contribution of immune effectors to viral load, we used uninformative prior distributions in these model fits.

We found strong support for liberating parameter constraints in the mechanistic model, with a striking decrease in DIC of ∼43 from CD8^+^ immune control model and a considerable improvement in fit (Figure 3). Consistent with the results from Granger regression and the constrained mechanistic model we found that the effect of CD8^+^ T cells on viral load was consistently negative, indicating that the net impact of CD8^+^ T cells in all macaques is to reduce viral load. In contrast, the effect of NK cells on viral load was found to be positive for all macaques, suggesting that in this data set NK cells can contribute to an increase in viral load. For B cells we found a positive effect on viral load in 6 animals and a negative effect in 5 animals (Table S2). To quantify the impact of intrinsic cell death and immune cell killing on viral load we calculated the change in log VL per hour attributable to each factor. We found a small decrease attributable to intrinsic cell death (median - 0.009 log SIV RNA/ml plasma/h), a larger decrease due to CD8^+^ T cells (median - 0.046), an increase due to NK cells (median +0.022) and a small impact for B cells (median +0.004). These estimates varied between animals but were highly consistent within animals across different time points (Table S3).

## Discussion

Different factors have been postulated to play a role in viral control in immunodeficiency virus infection, notably target cell limitation, CD8^+^ T cells, NK cells and B cells. As these factors are usually studied in isolation, their relative importance and in vivo relevance has not yet been quantified.

We used three different methods to assess the relative importance of target cells, CD8^+^ T cells, NK cells and B cells in determining SIV viral load in rhesus macaques. All three methods found that target cell limitation alone cannot explain SIV viral load dynamics and that including CD8^+^ T cells resulted in substantially better predictions. Two of the three methods make no assumptions about the mode of action of CD8^+^ T cells. In the third method (mechanistic modeling) we showed that our conclusions were the same whether we assumed a lytic or a non-lytic mode of action of immune effector cells [71]–[73]. Including NK cells or B cells did not result in a substantial improvement in predictive ability.

The finding that CD8^+^ T cells were significant predictors of viral load dynamics did not follow trivially from viral load driving CD8^+^ T cell expansion, as Granger analysis showed that viral load was not a strong predictor of CD8^+^ T cells. There are a number of possible reasons why viral load may be poorly predictive of CD8^+^ T cell dynamics. One explanation is that antigen only needs to be above a threshold to generate CD8^+^ T cells. Another is that the maximum number of specific CTLs is limited and that this limit is reached relatively early in infection so a possible effect of viral dynamics on CD8^+^ T cell dynamics will only be captured when simulating very early HIV infection.

Quantification of the contribution of different death terms showed that CD8^+^ T cell killing and intrinsic cell death were the main causes of productively infected cell death. We estimate that approximately 40% of infected cell death can be attributed to CD8^+^ T cell killing, consistent with our previous estimates of CD8^+^ T cell killing based on the strength of selection for escape variants (20–40% in HIV-infected humans [25], more in SIV-infected macaques [74]). This relatively small role for CD8^+^ T cells may explain the reported lack of an association between the rate of viral clearance and disease stage of the individual [75].

Surprisingly, NK cell numbers explain a large part of the variance in viral load but contribute little (5.7%) to infected cell death. Additionally, when we did not constrain immune cells to have a negative impact on viral load, the model fit was consistently optimized by a solution in which NK cells have a positive effect on viral load. This raises the question whether an alternative, detrimental role for NK cells in HIV should be considered. Consistent with this, several studies have found a positive correlation between inhibitory KIR3DL1-NK cell receptors and delayed disease progression [22], [37]. Additionally, we have found that inhibitory KIR receptors can enhance detrimental as well as protective CD8^+^ T cell responses (NK Seich al Basatena & B Asquith unpublished observations) possibly supporting a detrimental role for NK cells in some circumstances. One possibility is that NK cells recruit CD4^+^ T cell to the site of infection, thus supporting the establishment and spread of the infection. Alternatively, NK cells could support HIV-infection by IFNγ production leading to immune activation which has been implicated as a strong predictor of disease progression [76], [77]. In our data set both the total NK population as well as the Ki67^+^ NK population showed a significant correlation with the number of Ki67^+^ CD4 T cells (Spearman correlation coefficient R = 0.53 and 0.49, p = 6.57×10^−8^ and 5.3×10^−5^ respectively). Our unexpected finding that NK cells may increase viral load illustrates the value of combining an impartial “hypothesis-free” empirical approach with the more traditional approach of functional, mechanistic models.

In summary, we show that CD4^+^ target cells and CD8^+^ T cells are significant determinants and Granger-causal predictors of SIV viraemia. The main causes of infected cell death are immune (CD8, NK, B)-independent death (47%) and CD8^+^ T cell killing (42%). We find that NK cells and B cells play a very limited role in the control of viraemia being neither Granger causal predictors of viral load nor significant determinants of viral load in a mechanistic model and contributing little (both approximately 6%) to infected cell death. Of note we find evidence that the net impact of NK cells may be detrimental for the host and lead to an increase in viraemia.

## Methods

### Data

Animals were infected with SIV_mac251_ i.v. or SIV_mac239_ i.r. and blood taken once a week for the first four weeks after infection and subsequently every four or eight weeks up to 24 weeks after infection. Viral RNA levels in plasma were quantitated using real-time reverse transcriptase PCR. CD4^+^ T lymphocytes were defined as CD3^+^CD8^−^, CD8^+^ T lymphocytes as CD3^+^CD8^+^, NK cells as CD3^−^CD8^+^ and B cells as CD20^+^ lymphocytes. The density of these populations in peripheral blood was determined by multi-parameter flow cytometry. The fraction of proliferating CD4^+^ T cells, CD8^+^ T cells, B cells and NK cells was assessed by staining for the nuclear antigen Ki67, which is expressed by cycling cells. SIV-specific CTL activity against Env and Gag was determined after *in vitro* stimulation by ^51^Cr release assay. Seven of the SIV_mac239_-infected macaques received a recombinant herpes simplex virus (HSV) vaccine expressing the Envelope and Nef proteins of SIV_mac239_ prior to infection. The data for some of the macaques has previously been reported [69], [78]–[80]. A one-way analysis of variance (ANOVA) between treatment groups showed no significant differences in viral load and number or fraction of CD4^+^ T, CD8^+^ T, B or NK cells between the groups. Hence, animals were clustered into one group.

### Ethics statement

Animals were maintained in accordance with the guidelines of the Committee on Animals of the Harvard Medical School and the *Guide for the Care and Use of Laboratory Animals* [78].

### Granger causality (empirical model)

Granger causality examines whether the prediction of variable Y based on past (lagged) information of this variable could be improved by incorporating past information of another variable X. If the ability to predict Y is significantly improved by including past measurements of X, then X is said to have a weak-causal or “Granger-causal” influence on Y [81].

In this study bivariate Granger causality was implemented in the form of prediction of the current value of viral load V(t), based on lagged values of viral load V(t-τ) and lagged values of X(t-τ), where the explanatory variable X represents either target cells (T) or one of the immune populations, CD8^+^ T cells, CD20^+^ B cells or NK cells (E). For this analysis we only used 11 data sets that have at least of 6 data points. We tested for Granger-causality in those macaque data set using unequal time lags with lag-length based on available data, increasing with time since infection from 2 to 4 to 8 wks. 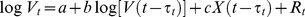
(1)


where τ_t_ is the lag at time t and R are the residuals or prediction errors. Implementation of a more complex model, explicitly incorporating the length of the time lags, did not change the results of the analysis (data not shown). To test if combination of variables can improve the prediction, the regression was extended to include past viral load and both past target and past effector populations. 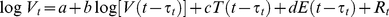
(2)


If there is no improvement of model accuracy this suggests the corresponding variable has no direct effect on log[V(t-τ)] [81]. Additionally, we reversed this analysis to address the question of Granger-causal influence in the opposite direction, i.e. if past viral load predicts future target or effector cell numbers.

### Mechanistic models

To optimise the use of available experimental data, minimise the number of parameters to be estimated and constrain the fits we followed the novel approach of Regoes *et al*. [66]. This approach is based on a dynamical model in which free virions (V) and infected target cells (T^*^) are described by deterministic ordinary differential equations.
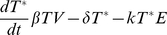
(3)

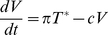
(4)


In this model uninfected cells (T) are infected by free virus at rate β to produce infected cells (T*) which die at rate δ and are cleared by immune effectors (E) at rate k. Free virus is produced by infected target cells at a rate π and cleared at rate c. We assumed a quasi-steady state between infected cells and free virus allowing us to eliminate Eqn 4. The number of target cells and effector cells, T(t) and E(t) were entered as empirical functions rather than predicted, putting an extra constraint on fitting the measured viral load dynamics, so the model reduces to
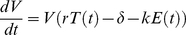
(5)


Where E(t) and T(t) are the empirical functions obtained by linear interpolation between data points. Parameter δ is the immune (CD8, NK, B)-independent death rate of infected cells; k is the rate of killing per effector cell per day and r (r = πβ/c) is the replication rate of the virus per target cell per day. Equation 5 can be solved to give:
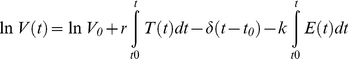
(6)


We refer to the version of the model (Eqn 6) in which the final term is omitted as the target cell limitation model; the full model is referred to as the immune control model. Immune effector populations were either CD8^+^ T lymphocytes, NK cells or B cells. We fitted the models to virus load measurements using both a Bayesian and a more conventional least squares regression approach. Additionally, to systematically test all possible combinations of immune effector populations the last term of equation 6 was repeated, data of the different effector populations were included and a separate k for each of the effector populations was estimated. We only had data on B cells for 11 of the 17 macaques, so models including the B cell populations were not fitted to all data sets.

Although CD8^+^ T cells are thought to control infection primarily via cytolysis there is also evidence for a non-lytic mode of action. We therefore also considered a non-lytic model for CD8^+^ T cells. In this model we assumed CD8^+^ T cell decrease the rate of infection of target cells.
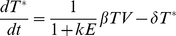
(7)


Replacing Eqn (3) with (7) gives:
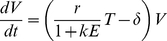
(8)


No analytical solution exists and, due to software limitations, we fitted this model using the conventional least squares regression approach only.

Although a cytotoxic role for B cells has been described, especially in acute infection, [82], [83] the main mode of B cell protection is thought to be via neutralizing antibodies. We modelled this by assuming that the number of B cells was proportional to the amount of neutralizing antibodies and built a model in which antibodies reduce the infection rate of uninfected target cells. 
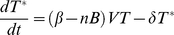
(9)


Replacing (3) with (9) and solving gives:
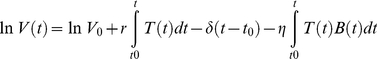
(10)


Where r = πβ/c and η = πn/c.

During primary SIV infection, total CD20^+^ lymphocyte population correlates with both disease progression and virus-binding antibodies [84] while total NK cell population significantly correlates with proliferation (0.612, p<0.01) which, in turn, parallels cytotoxicity [85], [86]. The available data enabled us to investigate the role of different immune cell populations and test which explains the viral load data best. For CD4^+^ T cell, B cell and NK cell populations we use both total populations and the proliferating Ki67^+^ fraction. CD8 T cells were variously defined as the total CD8^+^ T cell population, the Ki67^+^ fraction or the SIV-specific fraction represented by Env and Gag specific lytic units.

### Bayesian approach and MCMC

For the two models described above, parameters were estimated by fitting all data from all animals simultaneously but allowing parameters to vary from one macaque to another through random effects that were assumed to be normally distributed. A Bayesian approach was used to estimate the parameters. The Bayesian approach to population modelling is particularly useful as it allows the inclusion of prior knowledge in the form of informative prior distributions of parameter values. It integrates the prior information with the likelihood of the observed data to obtain a posterior distribution [87]. The joint posterior distribution of model parameters and data was explored by Markov chain Monte Carlo (MCMC) sampling [88]. Following Gelman and Rubin [89], 3 chains were run from different starting values until convergence, based on diagnosis of Brooks-Gelman-Rubin plots, was reached. Subsequently, the chains were run for a further 50,000 iterations and posterior distributions of each parameter, model fit and Deviance Information Criterion (DIC) were determined.

In the mechanistic models all priors were assumed to be normal and truncated to constrain the parameters to positive values. The mean and precision of the priors was based on estimates from the literature (Table 5). In the Granger analysis the parameters do not have a biological interpretation and so we choose uninformative priors with mean 0 for all parameters. Parameters can be the same for the whole population or allowed to vary between different animals, by using random effects. In order to restrict the number of effective parameters to the minimum needed we tested both options for all parameters but concluded that individual parameter values for each animal gave substantial better fits for all models, except the target cell model. All fits were performed in WinBUGS 1.4.3 (Imperial College and Medical Research Council, UK).

**Table 5:** Prior distributions of the parameters in the mechanistic models.

parameter	mean	precision	reference
δ	7.0	0.48	Markowitz *et al* 2003 [92]
r	0.21	0.001	Regoes *et al* 2004, Perelson *et al* 1996 [66], [93]
kCD8	0.0025	350	Based on Markowitz *et al* 2003 [92]
kNK	0.0058	150	Idem
kB	0.0035	250	Idem

All parameters were assumed to be positive and prior distributions truncated at 0. r =  production rate virus per target cell per week, δ =  death rate of infected cells per week, k =  killing rate per immune cell per week. In the immune control model the prior distribution of δ is changed to mean 3.5 and precision 0.25, in order to obtain a prior distribution of the total death rate (δ+kE) that is consistent with published data [92].

### Model inference/DIC

Significance of model fit was assessed by the Deviance Information Criterion (DIC), a Bayesian generalisation of the Akaike Information Criterion. Like the Akaike Information Criterion, the DIC is based on the goodness of fit penalized by the effective number of parameters or model complexity [90].

Model inference was based on the comparison between the DIC of two models (ΔDIC  =  DIC_1_-DIC_2_). As a rule of thumb for model inference Spiegelhalter *et al* [90] suggested that if models differ by only one or two DIC units then one cannot distinguish between the two models, if models differ by three to seven DIC units there is some support for the 1^st^ model but the 2^nd^ model is clearly better, or vice versa in the case of a negative ΔDIC. If the difference in DIC is greater than 10 essentially no support is found for the model with the higher DIC.

### Least squares regression approach

As an alternative, and more traditional, approach the model was also fit to the longitudinal data using non-linear least-squares regression defined in a global optimization algorithm (GlobalSolve from Maple ToolBox, MapleSoft, Canada). As opposed to the Bayesian approach where parameter estimates are described as random variables with a given distribution, this method provides point estimates for all parameters and the goodness of fit is described by the Sum of Squared Residuals. Comparison between models was based on this goodness of fit. For nested models model inference was based on an F-test on the individual data-sets followed by Fishers combined probability test. For non-nested models with an equal number of parameters we used a Wilcoxon sign test on the Sum of Squared Residuals.

For most models included in this study the conclusions reached by the two different approaches, Bayesian or least squares regression, agreed and so we do not discuss them both. We limit our discussion to the Bayesian approach and only include the least squares approach to the cases where the conclusions differ.

### Contribution to total death of infected cells

To determine the contribution of intrinsic cell death and immune cell killing to the total death rate of productively infected CD4^+^ T cells we divided the cell death attributable to intrinsic cell death (δ in equation 5) or attributable to one of the immune populations (k_x_E_x_(t)) by the total death rate at that time point. Intrinsic death rate and killing rates for each data set were determined in the model including all three death rates. Average contribution over time and standard deviation were determined. We also applied a more intuitive definition, by determining the increase in viral load in 1 hour when omitting one of the death terms relative to the increase in viral load found with the full model over the same time period. Conclusions of both methods were comparable.

### Partitioning of R^2^

To partition R^2^ according to the predictive power of each variable we used average stepwise regression [91]. In this method each variable's contribution to R^2^ can be estimated by entering each variable in the regression to viral load one by one and calculating the increment in R^2^ after each addition. Because the contribution of each variable is highly dependent on the order in which the variable is included in the equation, in average stepwise regression all possible orderings were considered and the average increment in R^2^ over all orderings was calculated. We looked at the contribution of past values of viral load, CD4^+^ T cell, CD8^+^ T cells, NK cells and B cells to explaining viral load variation in the total data set.

## Supporting Information

Table S1 Sum of Squared residuals of lytic and non-lytic CD8^+^ T cell model. Sum of Squared residuals of lytic and The non-lytic model (which has the same number of free parameters as the lytic model) gives a significantly improved fit to the data. This supports our conclusion that CD8^+^ T cells are important determinants of viral dynamics and motivates further studies into non-lytic mechanisms of CD8^+^ T cell control. Due to software limitations we were only able to fit the non-lytic model using the conventional least squares regression approach, for this reason the non-lytic model does not appear in Table 3 (however, its inclusion could only strengthen our conclusions as CD8^+^ T cells are already the best predictors of viral load dynamics using a lytic model).
(0.04 MB DOC)

Table S2 Fitted parameter values mechanistic model. Parameter values resulting from the fit of the mechanistic model including all three immune effectors without constraints on parameter ranges of the killing rates.
(0.04 MB DOC)

Table S3 Impact on viral load. Increase in viral load in 1 hour when omitting the indicated death term compared to the increase in viral load found with the full model over the same time period. To calculate the viral load parameter values resulting from the model fits are used. Increase in viral load is determined for each macaque at different time points at 1 week intervals.
(0.05 MB DOC)
