# Supplementary material for: Quantification of the Relative Importance of CTL, B Cell, NK Cell, and Target Cell Limitation in the Control of Primary SIV-Infection
Source: PLoS Comput Biol. 2011 Mar 3;7(3):e1001103. doi: 10.1371/journal.pcbi.1001103 (PMC3048377; doi:10.1371/journal.pcbi.1001103)
Supplement: Table S1 — Sum of Squared residuals of lytic and non-lytic CD8+ T cell model. Sum of Squared residuals of lytic and The non-lytic model (which has the same number of free parameters as the lytic model) gives a significantly improved fit to the data. This supports our conclusion that CD8+ T cells are important determinants of viral dynamics and motivates further studies into non-lytic mechanisms of CD8+ T cell control. Due to software limitations we were only able to fit the non-lytic model using the conventional least squares regression approach, for this reason the non-lytic model does not appear in Table 3 (however, its inclusion could only strengthen our conclusions as CD8+ T cells are already the best predictors of viral load dynamics using a lytic model). (0.04 MB DOC) [file pcbi.1001103.s001.doc]

| data set | Lytic | Non-lytic |
| --- | --- | --- |
| m285 | 39.94 | 10.72 |
| m137 | 29.50 | 1.69 |
| m159 | 50.28 | 19.16 |
| m342 | 29.55 | 29.52 |
| m178 | 5.03 | 0.70 |
| m410 | 7.01 | 6.14 |
| m198 | 48.07 | 6.07 |
| m325 | 38.88 | 5.34 |
| m260 | 84.85 | 9.49 |
| m265 | 48.15 | 35.22 |
| m330 | 30.19 | 4.33 |
| m112 | 28.58 | 3.57 |
| m363 | 64.48 | 2.27 |
| m364 | 51.27 | 9.28 |
| m83 | 32.40 | 5.68 |
| m85 | 65.14 | 24.23 |

**Table S1: Sum of Squared residuals of lytic and non-lytic CD8^+^ T cell model.** The non-lytic model (which has the same number of free parameters as the lytic model) gives a significantly improved fit to the data. This supports our conclusion that CD8^+^ T cells are important determinants of viral dynamics and motivates further studies into non-lytic mechanisms of CD8^+^ T cell control.

Due to software limitations we were only able to fit the non-lytic model using the conventional least squares regression approach, for this reason the non-lytic model does not appear in Table 3 (however, its inclusion could only strengthen our conclusions as CD8^+^ T cells are already the best predictors of viral load dynamics using a lytic model).
