# Supplementary material for: Quantification of the Relative Importance of CTL, B Cell, NK Cell, and Target Cell Limitation in the Control of Primary SIV-Infection
Source: PLoS Comput Biol. 2011 Mar 3;7(3):e1001103. doi: 10.1371/journal.pcbi.1001103 (PMC3048377; doi:10.1371/journal.pcbi.1001103)
Supplement: Table S2 — Fitted parameter values mechanistic model. Parameter values resulting from the fit of the mechanistic model including all three immune effectors without constraints on parameter ranges of the killing rates. (0.04 MB DOC) [file pcbi.1001103.s002.doc]

δ (wk^-1^)

Killing rate CD8^+^ T cells (cell^-1^.wk^-1^)

Killing rate B cells (cell^-1^.wk^-1^)

Killing rate NK cells (cell^-1^.wk^-1^)

Replication rate (cell^-1^.wk^-1^)

**Table S2: Parameter values** resulting from the fit of the mechanistic model including all three immune effectors without constraints on parameter ranges of the killing rates.
