# Supplementary material for: Quantification of the Relative Importance of CTL, B Cell, NK Cell, and Target Cell Limitation in the Control of Primary SIV-Infection
Source: PLoS Comput Biol. 2011 Mar 3;7(3):e1001103. doi: 10.1371/journal.pcbi.1001103 (PMC3048377; doi:10.1371/journal.pcbi.1001103)
Supplement: Table S3 — Impact on viral load. Increase in viral load in 1 hour when omitting the indicated death term compared to the increase in viral load found with the full model over the same time period. To calculate the viral load parameter values resulting from the model fits are used. Increase in viral load is determined for each macaque at different time points at 1 week intervals. (0.05 MB DOC) [file pcbi.1001103.s003.doc]

Impact of immune-independent cell death (log SIV RNA/ml plasma)

| data set | impact |
| --- | --- |
| 285.95 | 0.003 |
| 137.96 | 0.007 |
| 159.96 | 0.009 |
| 342.96 | 0.010 |
| 178.97 | 0.009 |
| 409.98 | 0.009 |
| 410.98 | 0.012 |
| 198.97 | 0.012 |
| 325.97 | 0.010 |
| 260.95 | 0.010 |
| 265.95 | 0.007 |

Impact of CD8^+^ T cells on viral load (log SIV RNA/ml plasma)

Impact of B cells on viral load (log SIV RNA/ml plasma)

Impact of NK cells on viral load (log SIV RNA/ml plasma)

**Table S3: Impact on viral load.** Increase in viral load in 1 hour when omitting the indicated death term compared to the increase in viral load found with the full model over the same time period. To calculate the viral load parameter values resulting from the model fits are used. Increase in viral load is determined for each macaque at different time points at 1 week intervals.
